# PCM-SABRE: a platform for benchmarking and comparing outcome prediction methods in precision cancer medicine

**DOI:** 10.1186/s12859-016-1435-5

**Published:** 2017-01-17

**Authors:** Noah Eyal-Altman, Mark Last, Eitan Rubin

**Affiliations:** 1 Shraga Segal Department of Microbiology and Immunology, Faculty of Health Sciences, Ben-Gurion University of the Negev, Beer-Sheva, 84105 Israel; 2 Department of Software and Information Systems Engineering, Ben-Gurion University of the Negev, Beer-Sheva, 84105 Israel

**Keywords:** Breast cancer, Data mining, Reproducible research

## Abstract

**Background:**

Numerous publications attempt to predict cancer survival outcome from gene expression data using machine-learning methods. A direct comparison of these works is challenging for the following reasons: (1) inconsistent measures used to evaluate the performance of different models, and (2) incomplete specification of critical stages in the process of knowledge discovery. There is a need for a platform that would allow researchers to replicate previous works and to test the impact of changes in the knowledge discovery process on the accuracy of the induced models.

**Results:**

We developed the PCM-SABRE platform, which supports the entire knowledge discovery process for cancer outcome analysis. PCM-SABRE was developed using KNIME. By using PCM-SABRE to reproduce the results of previously published works on breast cancer survival, we define a baseline for evaluating future attempts to predict cancer outcome with machine learning. We used PCM-SABRE to replicate previous work that describe predictive models of breast cancer recurrence, and tested the performance of all possible combinations of feature selection methods and data mining algorithms that was used in either of the works. We reconstructed the work of Chou et al. observing similar trends – superior performance of Probabilistic Neural Network (PNN) and logistic regression (LR) algorithms and inconclusive impact of feature pre-selection with the decision tree algorithm on subsequent analysis.

**Conclusions:**

PCM-SABRE is a software tool that provides an intuitive environment for rapid development of predictive models in cancer precision medicine.

**Electronic supplementary material:**

The online version of this article (doi:10.1186/s12859-016-1435-5) contains supplementary material, which is available to authorized users.

## Background

Predicting the outcome of cancer from gene expression data is a clinically important, computationally challenging task. For example, early-stage, estrogen-receptor-positive, HER2-negative breast cancer patients that are considered to be at low risk for recurrence can avoid chemotherapy, while patients at high or intermediate risk are treated with aggressive (and harmful) chemotherapy [1].

Efforts to stratify patients by risk of recurrence in other tumor types, and the ability to stratify patients by overall chances of survival are not as advanced. Moreover, the relative success in risk stratification for breast cancer patients has been challenged [2], proposing that it in fact stratifies patients into tumor subtypes, which can be achieved with much simpler tests.

As a result, a large number of papers have been published and are still being published where gene expression data is analyzed in order to construct models that predict cancer survival or cancer recurrence. Much of these efforts are concentrated on breast cancer, the second most commonly diagnosed cancer among American women (besides skin cancer) [3]. About 1 in 8 U.S. women (about 12%) will develop invasive breast cancer over the course of her lifetime, and similar rates are reported worldwide [4]. Breast cancer is an attractive domain for risk stratification as it is estimated that resection is a sufficient treatment for 70 to 80% of the patients, while the remaining patients will develop advanced metastatic lesions, which are largely impossible to cure [5]. Aggressive chemotherapy will reduce the chance of advance metastasis for those patients that would have advanced at the expanse of harmful an un-necessary therapy for those who would note. Thus, great efforts have been invested in stratifying patients’ risk of recurrence [6].

Due to the importance of risk stratification in breast cancer, combined with its relatively high abundance, breast cancer is the type of tumor for which expression profiles of newly diagnosed patients are most abundant. Several works have been published that apply machine-learning techniques to this data for predicting cancer survivability (for example [7] and [8]). Unfortunately, we found it quite challenging to directly compare these works for the following reasons:

1. Incomplete specification of critical stages in the process of knowledge discovery, such as feature selection.
2. Differences in the measures used to evaluate models performance. Some only provide the overall accuracy of the proposed classifier, some offer only the Area Unser the Curve (AUC), while others provide no statistical measures and only present the Kaplan-Meier charts that visualize the survival curves based on predicted classes.
3. Different studies apply different inclusion/exclusion criteria with little or no overlaps between the patients considered.

Incomplete documentation of the analytic process is a common cause for irreproducibility of published results. We conclude that there is a need for a platform that would allow researchers to describe their analytic work in the field of risk stratification for cancer patients in a reproducible way that can be used for further investigation. Such a platform should allow to replicate previous works and to methodologically evaluate the impact of alterations in one or more stages of the knowledge discovery process on its performance in the task of cancer survival prediction. Such a tool can help to understand and compare the current state of predictions for breast cancer, and if applied to new cancer types, to prevent the “Tower of Babel” situation that has emerged for breast cancer.

## Implementation

**Fig. 1:**
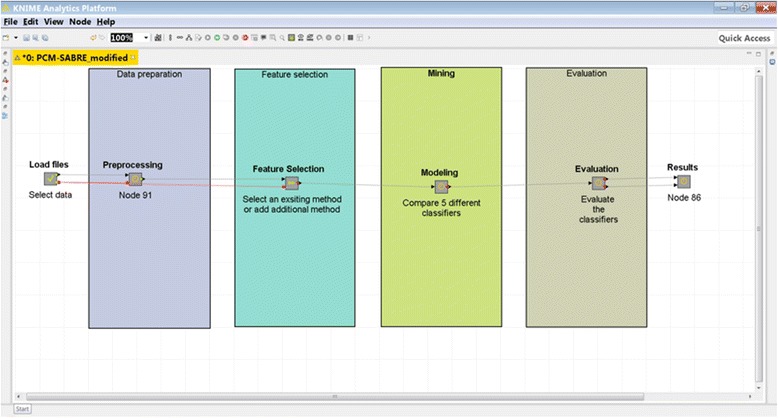
Screenshot of PCM-SABRE

We developed a platform that allows replicating, comparing and improving knowledge discovery pipelines for cancer survival predictions, and demonstrate its applicability for Breast Cancer (Fig. 1). PCM-SABRE (Precision Cancer Medicine - Survival Analysis Benchmarking, Reporting and Evaluation), was developed using KNIME (Konstanz Information Miner; [9]). KNIME is a modern, flexible and intuitive open-source data analytics platform that allows performing sophisticated statistics and data mining analysis to develop, among other things, predictive models. We chose KNIME since it is a popular, user-friendly software that does not require programing knowledge. Its node-based workflow structure allows easily assessing the impact of changing one knowledge discovery step (for example, data mining algorithm) on the predictive performance without changing any other steps of the workflow. Another major benefit of KNIME is the ability to create new nodes, this feature is particularly useful when a researcher is interested in integrating a new method he developed into an existing KNIME workflow.

**Fig. 2:**
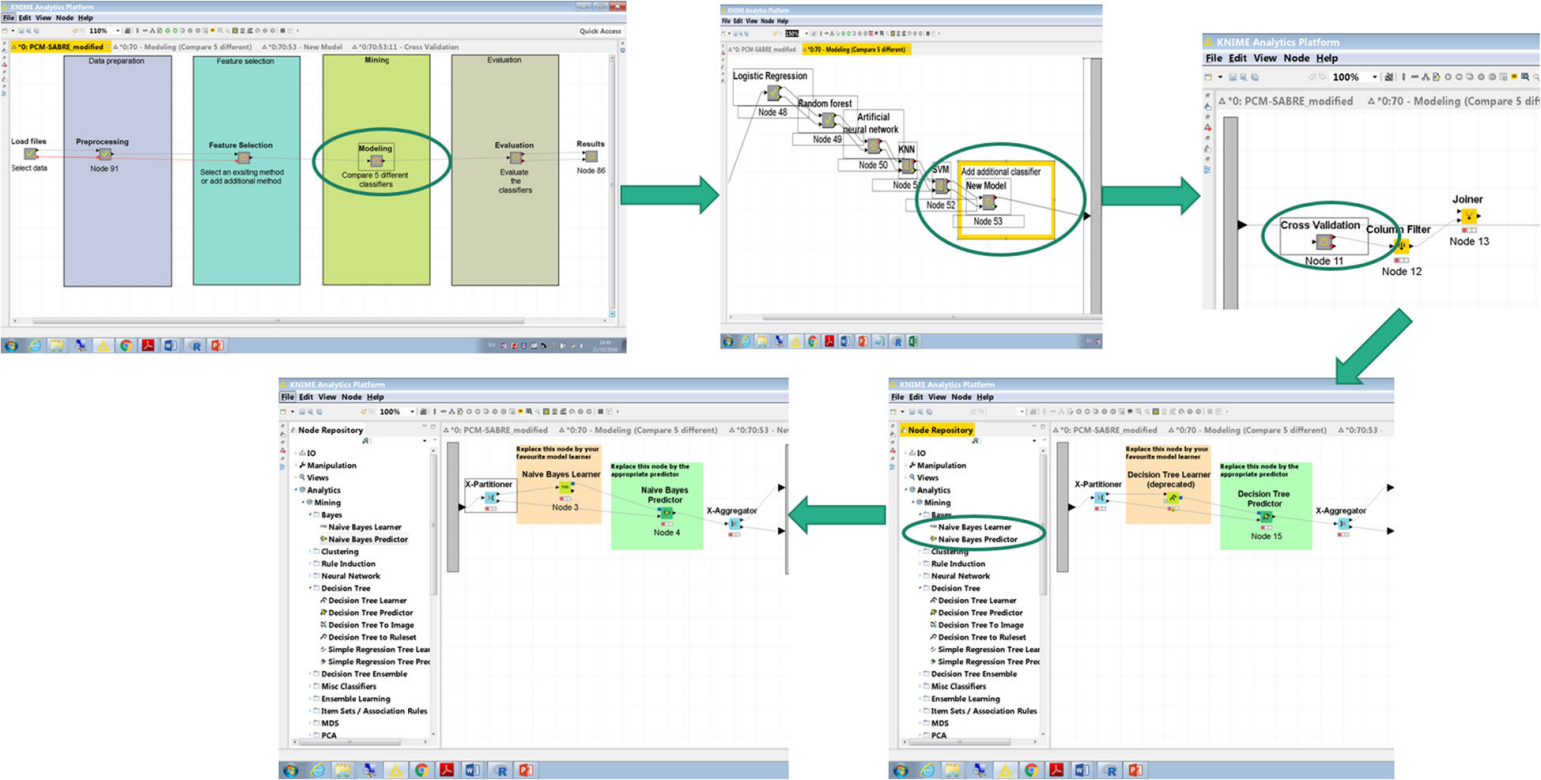
Demonstration of drag-and-drop model replacement (Naïve Bayes instead of decision tree)

We designed PCM-SABRE workflow according to the common steps of knowledge discovery in data. First, the user can use a supplied dataset or load a new dataset. The dataset has to be a csv file in the form of a table in which the rows represents the patients and the columns represents clinical data, gene expression data or any other types of variables; the dependent variable can be binary or continuous (it will be transformed into a binary variable) and need to represent survival time (for example, Relapse-free-survival time or death time). The second Meta-node is the preprocessing step, where a binary dependent variable is created and patients with missing data or censored survival information are being filtered. We chose to use a default threshold of 5 years in order to split the continues survival variable into HIGH (t < 5 years) or LOW (t ≤ 5 years) risk, but this threshold is an input parameter that can be changed in a way that will be explained later. Missing values imputation is performed using random forest classification that builds a model using the non-missing rows and predicts the variable value for the missing rows. The default version of PCM-SABRE allows selecting patients according to their ER status and Lymph node status but the “Select Patients” Meta-node is optional and can be easily modified to meet other inclusion/exclusion criteria. The third Meta-node is the feature selection step, where the users can choose between two methods of feature selection (information gain or ANOVA) or add another feature selection method (from the available nodes in KNIME, using scripting or external tools). The fourth Meta-node is the modeling step, where we offer a choice of 5 well-known and relevant classifiers. The methods included in the out-of-the-box basic version of the workflow are described in Table 1. It should be noted that thanks to the design of KNIME, adding additional Modeling and Feature Selection methods involves just dropping additional nodes in the appropriate Meta-nodes and connecting them by drag-and-drop using the existing methods as templates. Our experience with experimental biologists suggests that any oncology researcher without programming capabilities can achieve this with little or no special training, Fig. 2 illustrates how the user can easily and quickly add additional classifier to the workflow: (1) double-click modeling → new model → cross-validation (2) delete the decision tree learner and predictor (3) choose from the Node Repository another learner and predictor nodes and drag-and-drop them instead of the deleted nodes (4) connect the X-Partitioner node Training data output into the Learner node input, connect the Learner node PMML output into the PMML input of the Predictor node, connect the Predictor node to the X-Aggregator node and connect the X-partitioner Test data output to the Predictor node. The fifth Meta-node is the evaluation step, which calculates the performance measures of different models (among them the accuracy and the Area under the ROC). An important feature of PCM-SABRE is a csv file (flow_variables.csv) that allows the user to control some default input parameters without the need to change these parameters inside the specific KNIME nodes. The controlled input parameters are: (1) Feature selection method (default = infoGain), ER status (default = all patients), Lymph node status (default = all patients) and the threshold for the binary survival variable (default = 5 years). Changing and adding another input parameter is simple and only requires filling cells in excel. Additional details on how to use PCM-SABRE can be found in the User Manual.

**Table 1:** Machine learning methods available in PCM-SABRE

Meta-node		Method	KNIME node	Default parameters
1.1	Select patients	Estrogen Receptor status (ER)	R script	
1.2	Select patients	Lymph Node status (LN)	R script	
2.1	Feature Selection	Information Gain (InfoGain)	InformationGainCalculator (Community node – Palladian)	Top 100 ranked
2.2	Feature Selection	ANOVA	One-way ANOVA	include genes with *p*-value < 1.0E-6
3.1	Modeling	Logistic Regression (LR)	Logistic (3.7) (Weka node)	Ridge = 1.0E-8,
3.2	Modeling	Random Forest (RF)	Random Forest Learner	Split criteria = Information Gain Ratio, Number of models = 350
3.3	Modeling	Artificial Neural Network (ANN)	PNN Learner (DDA)	Theta Minus = 0.2, Theta Plus = 0.4
3.4	Modeling	K-Nearest Neighbors (KNN)	IBK (3.7) (Weka node)	KNN = 15
3.5	Modeling	Support Vector Machine (SVM)	SVM Learner	Kernel = RBF, sigma = 0.2

PCM-SABRE output includes, for each combination of a feature selection method with a classification algorithm (1) performance measures, (2) ROC analysis and (3) list of ranked features.

## Results

We developed PCM-SABRE (available as Additional file 1) as a software system that allows to compare and improve expression-based predictive models of cancer patients. We used PCM-SABRE to replicate previous work that describe predictive models of breast cancer recurrence, and evaluated the performance of all possible combinations of feature selection methods and data mining algorithms that was used in either of the works.

### Using PCM-SABRE for replicating a previous work that utilizes machine learning to induce outcome prediction models

We first demonstrate the value of PCM-SABRE to investigators implementing new machine learning pipelines for breast cancer recurrence prediction by replicating the work of Chou et al. [10]. Our analysis reconstructs the paper to the best of our ability, with the following exceptions. We use KNIME rather than the original software (Clementine 10.1) and we use as input data a more current compendium of expression data (will be called Györffy dataset for the rest of this paper) [7]. The dataset is available for download here [11]. The Györffy dataset originally contained 1809 examples (breast cancer patients) and 22,216 features (clinical features and probes expression level). A binary class attribute was created indicating whether the cancer recurred within 5 years or not.

**Fig. 3:**
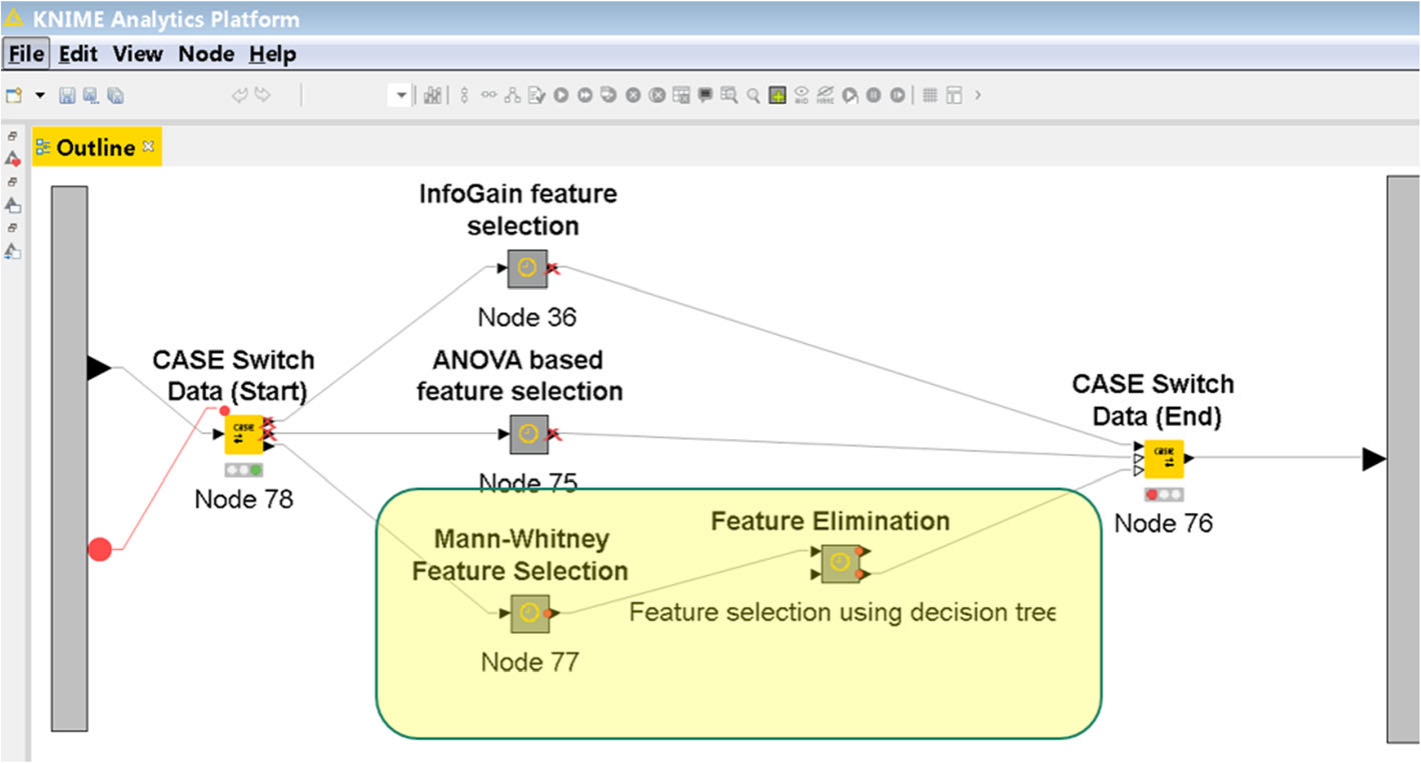
Modification of the feature selection Meta-node in order to replicate Chou et al. work

To best reproduce the original work, we made the following modifications to the default out-of-the-box KNIME pipeline:

1. A preprocessing step was added that reproduces the preprocessing performed in the original paper. This step was conducted with a specialized R script written for this purpose. In this step, features were transformed from probe to gene level. After the transformation, the dataset contained 13,725 features.
2. In the preprocessing Meta-node, we removed lymph node positive patients and patients with follow-up time of less than 5 years (1219 patients remained).
3. Two new feature selection methods were added to the feature selection Meta-node (Fig. 3):
  - The Mann–Whitney *U* test was used for decreasing the number of genes from 13,725 to 100 exactly as described in [10]. The Mann–Whitney U non-parametric test, which is also known as the Wilcoxon rank sum test, tests for differences between two groups on a single, ordinal variable with no specific distribution [12]. The *U* statistic of each group is calculated as a difference between the actual sum of ranks of the group observations and the sum expected value under the null hypothesis that the distribution of the ordinal variable in both groups is equal. More details are available in [10].
  - A compound selection method was added, in which the results of the DT algorithm were used to determine which features will be retained for PNN and LR analysis.
    DA (Decision tree + Probabilistic neural network) DT + PNN → DA
    DL (Decision tree + Logistic regression) DT + LR → DL

The classification performance results from PCM-SABRE and from the original paper are compared in Table 2. In contrast to the original work, PCM-SABRE reports that LR has the best performance. Moreover, both show a different trend when adding the DT feature selection methods. It is worth noting that the estimated accuracy reported by PCM-SABRE is higher than in the original work. This may be because a different dataset was used for the analysis.

**Table 2:** Predictive power (in terms of percent accuracy) of several feature selection methods combined with different classification models. AUC results are shown in brackets

Prediction model	PCM-SABRE pipeline			Chou et al. [10]
Feature selection	InfoGain	ANOVA	MW *U* test	MW *U* test
RF	76.52 (NA)	77.70 (NA)	76.10 (NA)	NA
LR	76.27 (73.0)	66.55 (62.49)	75.68 (70.95)	64.12 (58.96)
PNN	76.52 (74.09)	76.27 (75.21)	74.58 (72.32)	69.54 (63.88)
KNN	75.76 (67.78)	75.34 (68.48)	76.10 (70.30)	NA
SVM	72.64 (NA)	72.64 (NA)	72.64 (NA)	NA
DT	70.19 (60.59)	68.07 (61.53)	64.44 (57.34)	63.45 (56.90)
DL	NA	NA	75.34 (71.71)	68.90 (61.66)
DA	NA	NA	75.51 (72.23)	65.91 (61.65)

### Using PCM-SABRE for optimizing and improving breast cancer outcome prediction

For the task of breast cancer outcome prediction, we used again the dataset published by Györffy et al.and conducted the preprocessing steps maintained above. Table 2 summarizes the performance of all combinations of feature selection methods and classification algorithms. LR, PNN KNN and DT performed better combined with the InfoGain feature selection method, in terms of Accuracy but not in terms of AUC. RF performed better combined with the ANOVA feature selection method and achieved the highest Accuracy (77.70%).

## Discussion

We developed an intuitive platform for comparing machine learning pipelines for survival prediction. To demonstrate the usefulness of our tool, we first show that with minimal modifications, PCM-SABRE can be used to reconstruct machine learning pipelines from the literature, and to explore the impact of changes in the process (such as adding sequential feature selection) on its performance. We reconstructed the work of Chou et al. similarly observing the superior performance of PNN and LR over DT, but the impact of feature pre-selection with the DT algorithm on subsequent algorithm was inconclusive. These results reinforce the need for a platform like PCM-SABRE that would allow more reliable comparison between studies and reproducible results.

To further explore the usefulness of PCM-SABRE, we used it to methodologically explore various combinations of feature-selection/modelling algorithms. As expected, some algorithms perform better than others. However, we find that for the particular task of inducing a predictive model for breast cancer survival, in terms of Accuracy, information gain outperforms ANOVA for feature selection, with 4 out of 6 algorithms that were tested and achieved similar performance in two additional algorithms.

These results demonstrate the two main uses we propose for PCM-SABRE. First and foremost, future attempts to improve survival prediction can be reported using PCM-SABRE. This would ensure reproducibility of the analysis, as KNIME allows to bundle the input data with the algorithm. By publishing executable description of the process, the users will be able to run exactly the same pipeline, and even more importantly, the users will be able to understand and evaluate the particular contribution of each step in the process by changing it and observing the impact on model quality.

The other use we propose for PCM-SABRE is optimization of predictive models. Using KNIME it is straightforward to consider the impact of changing each step in the model induction process, and within the PCM-SABRE framework, the results are directly comparable. The ability to keep all other steps constant or to evaluate different combinations can allow non-experts to optimize their predictive models while ensuring the resulting process can be intuitively communicated to others.

Nowadays, more and more researchers who study breast cancer recurrence risk prediction specifically and researchers who study cancer outcome prediction in general, are increasingly using data mining and machine learning methods. In order to make a step forward in this field, the community has to put a greater emphasis on reproducible research. As we already maintained, as of today, it is almost impossible to compare between different “gene signature” papers that are being published. We believe that if researchers will implement their data analysis process on PCM-SABRE and will make their workflow available as an additional file, it will benefit everybody and will cause the prediction models and the gene lists that accompany them to be more reliable. Sharing KNIME workflow is very easy, KNIME allows to save the workflows with or without the input data file and a simple compression software will enable to publish the entire KNIME folder as a single file. The researcher can also add a screenshot of KNIME to a paper (maybe instead of the “usual” figure that describes the data analysis process).

Clearly, PCM-SABRE can be implemented with other intuitive pipeline development systems. RapidMiner [13] is a popular machine learning environment that can also be used for this purpose. RapidMiner is very similar to KNIME, both software tools are visual environments for predictive analytics, both are available for Windows, Mac and Linux and both offers online help forums, documentation and tutorials. Although RapidMiner is ranked higher in list of the top Analytics/Data Science Tools 2016 according to KDnuggets (5 vs. 9) [14], KNIME has a large customer base in the life sciences sector (bioinformatics and Next Generation Sequencing extensions can be found here [15]). In addition, we believe that KNIME is more intuitive and provides a “softer landing” for cancer researchers who are unskilled in programming and who are interested in sharing their data analysis workflow with other researchers. Other tools also exist, such as the WEKA workspace [16]. However, these are not sufficiently intuitive for untrained users. The features of KNIME which we think make it most attractive for this purpose are the ability to wrap critical parts of the process in metanodes, the strong branching and looping capability that supports evaluating alternative methods in parallel, and the ability to pass parameters to the pipeline, as a way to enhance user control without requiring a detailed editing of many nodes. We thus conclude that while PCM-SABRE can be implemented with other machine-learning platforms, KNIME offers a user-friendly yet powerful solution for this purpose.

The approach we present here is not unique to survival prediction from expression data: in principle, PCM-SABRE can also be used for developing other predictive models. However, as other projects may emphasize other steps in machine learning (e.g. feature extraction), more work is required to adapt PCM-SABRE for other tasks.

## Conclusions

PCM-SABRE is a software tool that provides an intuitive environment for a rapid development of predictive models in cancer precision medicine. It allows to easily define a data source and to consider alternative ways to conduct the main steps of the prediction process. The resulting pipeline can be shared with others in an intuitive yet executable way, which will improve, if adopted by other investigators, the comparability and interpretability of future works attempting to predict patient survival from gene expression data.

## Additional file

Additional file 1: PCM-SABRE Library. PCM-SABRE KNIME workflow. (RAR 45850 kb)

## Abbreviations

AUC: Area under the curve
DA: Decision tree for attribute selection and artificial neural network for classification
DL: Decision tree for attribute selection and logistic regression for classification
DT: Decision tree
ER: Estrogen receptor
InfoGain: Information gain
KNN: K-nearest neighbors
LN: Lymph node
LR: Logistic regression
PNN: Probabilistic neural networks
RF: Random forest
SVM: Support vector machine
